# *Cytauxzoon felis*: An Overview

**DOI:** 10.3390/pathogens12010133

**Published:** 2023-01-13

**Authors:** Yvonne M. Wikander, Kathryn E. Reif

**Affiliations:** Department of Diagnostic Medicine/Pathobiology, Kansas State University, Manhattan, KS 66506, USA

**Keywords:** *Amblyomma americanum*, cytauxzoonosis, *Dermacentor variabilis*, domestic cat, merogony, schizogony, sporogony, sporozoites

## Abstract

*Cytauxzoon felis* is a tick-transmitted, obligate, hemoprotozoal, piroplasmid pathogen of felids and the causative agent of cytauxzoonosis. It has a complex life cycle which includes a tick as its definitive host and a felid as its intermediate host. Since its first description in 1976, *C. felis* infections of felids have been reported in several southeastern and south-central U.S. states, overlapping with the ranges of its two known biological vectors, *Amblyomma americanum* (Lone star tick) and *Dermacentor variabilis* (American dog tick). Infected felids demonstrate disease as either an acute, often-fatal, infection, or a subclinical carrier infection. To develop effective *C. felis* transmission control strategies, the incidence of acute cytauxzoonosis, patient risk factors, the role of domestic cat carriers, and ecological variabilities need to be investigated further. Of equal importance is communicating these strategies for high-risk cat populations, including recommending year-round use of an acaricide product for all cats that spend any time outdoors. More studies are needed to further identify factors affecting *C. felis* and other *Cytauxzoon* spp. infection, transmission, disease progression, and treatment options and outcomes within the U.S. and globally. Here we provide an overview of *C. felis* highlighting its lifecycle within its definitive host, transmission to its intermediate host, symptoms and signs providing evidence of transmission, definitive diagnosis, current treatment and prevention strategies, and future considerations regarding this condition.

## 1. Introduction

To date, five *Cytauxzoon* spp. have been identified worldwide: *Cytauxzoon felis* in the Americas, *Cytauxzoon europaeus*, *Cytauxzoon otrantorum*, and *Cytauxzoon banethi* in Europe, and *Cytauxzoon manul* in Asia [1,2]. Although all five species are closely related, this article is intended as an overview or description of the *Cytauxzoon felis* organism found in the continental United States (U.S.), including its history, phylogenetic classification, anatomy, complex life cycle, and the repercussions of its passage through its definitive and intermediate hosts. Only limited epidemiologic data on the detection of *C. felis* infection in felids in the U.S. is presented in this overview. Readers interested in a thorough review of where feline cytauxzoonosis has been identified since its initial description in 1948, including felids infected by *Cytauxzoon* spp. found in Europe, Asia, and the Americas, are referred to two excellent review articles: Wang et al. 2017 [2] and, Reichard et al. 2021 [3].

**Figure 1:**
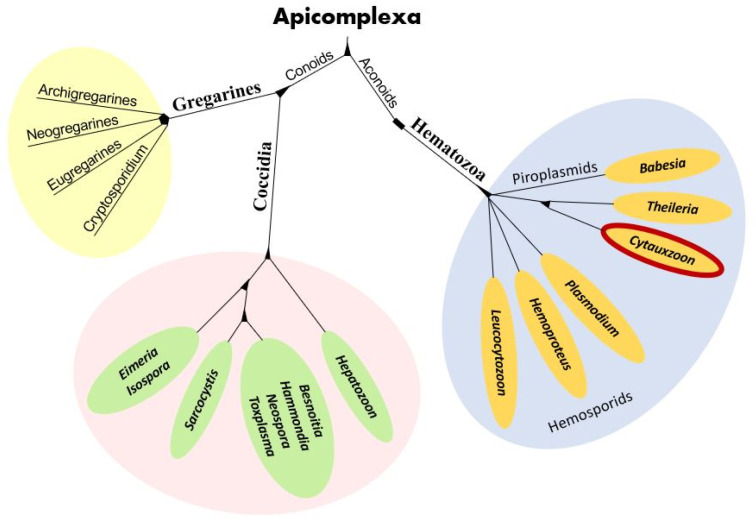
Apicomplexa phylogenetic tree with the genus *Cytauxzoon* (circled in red) within the family Theileriidae, order Piroplasmida, subclass Hematozoa, and phylum Apicomplexa.

*Cytauxzoon* organisms are apicomplexan protozoa in the subclass Hematozoa, order Piroplasmida, and family Theileriidae (Figure 1) [2]. The microscopic intra-erythrocytic pathogens of the *Cytauxzoon*, *Babesia*, and *Theileria* genera are often called piroplasms due to their 1–2-micron diameter pear-shaped or circular (signet ring) appearance [4]. *Cytauxzoon felis* is an obligate, hemoprotozoal, piroplasmid pathogen of felids and the agent of cytauxzoonosis, an often-fatal disease of domestic cats residing in the southeastern and south-central U.S. [3,5]. The history of *C. felis*, its phylogenetic placement, anatomy, and complex life cycle has been studied for over four decades with many questions still unanswered.

## 2. History

The *Cytauxzoon* genus was first described in 1948, when W.O. Neitz and A.D. Thomas characterized a *Theileria*-like piroplasm that underwent schizogony in histocytes of an African duiker (*Sylvicapra grimmia*) from South Africa [6]. Nearly thirty years later, *Cytauxzoon felis* was first described by J.E. Wagner of the University of Missouri when he characterized a *Cytauxzoon*-like piroplasm in four cats that suffered fatal disease outcomes from 1973 to 1975 [7]. Because *Cytauxzoon* organisms had previously only been described in African ungulates, the Plum Island Animal Disease Center of the United States Department of Agriculture (USDA) and the Animal and Plant Health Inspection Service (APHIS) endeavored to determine if the organism was a foreign disease and whether or not it posed a threat to the U.S. livestock industry [8]. To help address this question, over 500 cats were experimentally infected with *C. felis* in over 100 serial passages, and the course and outcome of the disease was studied [8]. Their findings suggested that the disease was: (1) largely fatal for domestic cats, and (2) unlikely to be a foreign animal disease, thus not a concern for U.S. food production. Since that time, molecular diagnostic methods suggest that the organism found in the African duiker was likely a *Theileria* species [9]. The potential for interspecies transmission of *C. felis* was investigated further when four domestic livestock species, nine lab animal species, and 17 wildlife species were experimentally inoculated with blood or tissue homogenates from euthanized domestic cats with confirmed acute cytauxzoonosis [10]. One of the bobcats in the study developed clinical signs while another bobcat and two sheep developed low-grade persistent parasitemia without clinical signs. No studies have investigated whether sheep with parasitemia can act as a competent host to infect the tick vector of this disease. Since its initial description in the U.S., *C. felis* has been identified in a variety of felid species within the U.S. and South America [2,3].

## 3. Phylogeny

In the past, taxonomic classification of organisms like *C. felis* was based on phenotypic similarities, but with the advent of molecular assays, genetic similarities have taken a greater role in phylogenetic organization [11]. Several genetic-based phylogenetic studies have been performed to flesh out the taxonomic organization of the order Piroplasmida within the phylum Apicomplexa. These have included the 18S gene, C1A-cysteine proteinase (a cell invasion-egress enzyme) gene, and a linked 18S ribosomal subunit-cox1 mitochondrial amino acid sequence evaluation [11,12,13]. The results of these studies suggest five distinct Piroplasmida groups: (1) *Babesia* sensu strico, (2) *Theileria* and *Cytauxzoon*, (3) *Theileria equi*, (4) *Babesia conradae*, and (5) *Babesia microti* spp. [11]. The taxonomic classification of other *Cytauxzoon* spp. found in Europe (*C. europaeus*, *C. otrantorum*, and *C. banethi*) and Asia (*C. manul*) using the 18S rRNA genes have determined all five species are related with <2% identity difference between them [1,2]. Although the genetic difference between the three European *Cytauxzoon* spp. and *C. manul* is only 0.39%, a recent study suggests they are closely related but separate species [1].

## 4. Anatomy

**Figure 2:**
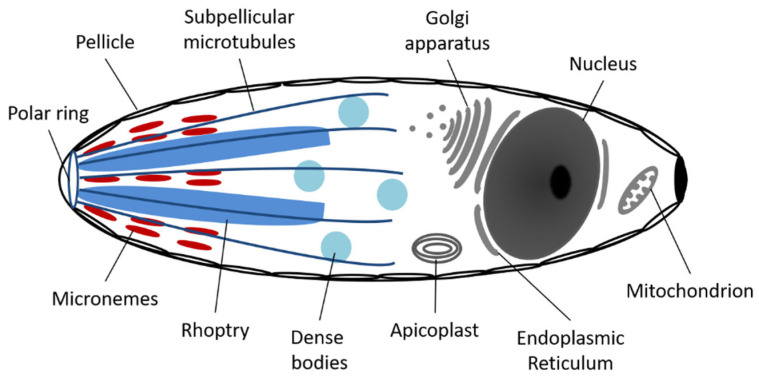
Anatomy of an apicomplexan sporozoite.

Like all Apicomplexa, each *C. felis* organism contains a nucleus, endoplasmic reticulum, Golgi apparatus, a mitochondrion, an apicoplast, and a pellicle with an apical complex (Figure 2) [14]. The first four organelles have functions much like those in other unicellular or multicellular eukaryote organisms, whereas the apicoplast and apical complex are unique to members of this phylum. The apicoplast contains its own circular DNA and is surrounded by triple or quadruple membranes, supporting an endosymbiotic origin. Although the exact function of this organelle is unclear, it has been proposed that it may take part in several potential pathways including the synthesis of fatty acids, heme breakdown, amino acid synthesis, isoprenoid precursors, and/or iron-sulfur clusters [12]. More studies are needed to fully elucidate the function of apicoplasts in these organisms.

The apical complex and pellicle consists of up to seven distinct cytoskeletal components, each with a specialized function (Figure 2) [4,12]. The outer membrane and subpellicular microtubules of the pellicle provide a variably elastic cell shape to the organism [15]. The polar ring, made up of microtubular bands at the apical end of the organism, acts as an organizing center for the subpellicular microtubules and gives the cell polarity. The small dense bodies called micronemes secrete adhesive proteins allowing the hemoprotozoan to move along the host cell membrane in a gliding motion, just prior to penetration [14,16]. Rhoptries, large saccular electron dense bodies, and dense body vacuoles secrete dissolving enzymes to enable penetration into the host cell [4,12]. The final apical complex structure, lacking in all Hematozoans including *C. felis*, is the conoid, a spiral cone of microtubules associated with the polar ring, used as a feeding tube for cellular vampirism in some apicomplexans (not depicted in Figure 2) [12].

The one mitochondrion found in each apicomplexan organism generally contains multiple copies of short (6-kb) circular or linear strands of DNA (miDNA) [14]. The number of mitochondrial genome copies per mitochondrion in each apicomplexan species varies from a handful to over 100 copies each, which may further vary based on the apicomplexan life stage. This redundancy may be a survival mechanism developed to maintain nucleic acid sequence integrity by allowing the disposal of miDNA damaged by reactive oxygen species (ROS) or harmful mutations [17]. The number of mitochondrial genome copies per *C. felis* mitochondrion has yet to be determined. Interestingly, apicomplexan miDNA contains genes for only three electron transport chain (ETC) proteins: cytochrome b (Cytb), cytochrome c oxidase subunit I (Cox1), and cytochrome c oxidase subunit III (Cox3) [14]. As such, they are missing the necessary genes for at least two of the needed mitochondrial ETC subunits to perform oxidative phosphorylation and may derive all their energy via anaerobic glycolysis [18]. It has been proposed that mitochondrion ETC functions in these organisms include: (1) providing an electron sink for ubiquinone-dependent dehydrogenase used in cell metabolism for mitochondrial protein degradation, (2) maintaining a transmembrane gradient for metabolite and protein transport, and/or (3) reducing ROS. Additional studies are needed to evaluate the specific function of this organelle in apicomplexan organisms.

## 5. Life Cycle

### 5.1. Summary

**Figure 3:**
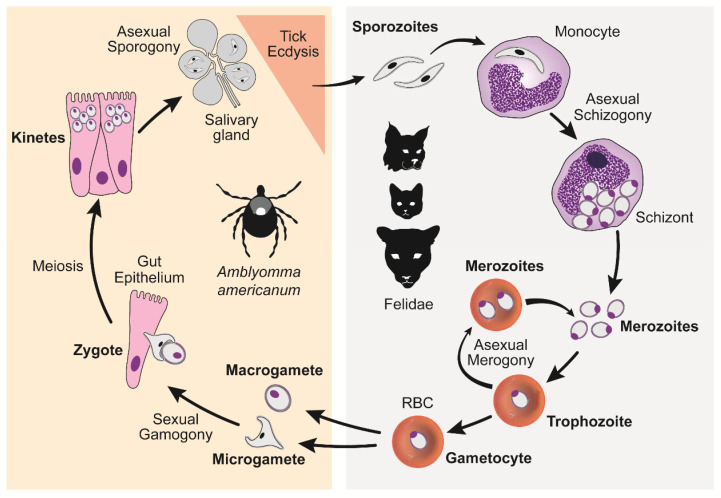
*Cytauxzoon felis* lifecycle demonstrating asexual reproduction within the host felid (**right panel**) and both sexual and asexual reproduction within the tick transmission vector (**left panel**). (Wikander Y.M.; Anantatat T.; Kang Q.; Reif K.E. Prevalence of *Cytauxzoon felis* Infection-Carriers in Eastern Kansas Domestic Cats. *Pathogens* **2020**, *9*, 854. https://doi.org/10.3390/pathogens9100854. PMID: 33092245; PMCID: PMC7594093) [5].

*Cytauxzoon felis* has a complex lifecycle that starts, for the feline host, with the injection of *C. felis* sporozoite-laden saliva from a feeding tick [4] (Figure 3). Each sporozoite invades a monocyte and undergoes asexual division (schizogony) resulting in hundreds if not thousands of ring-shaped merozoites being released into the blood when the monocyte ruptures. Each merozoite, or piroplasm, invades an erythrocyte and either develops into a non-replicating macro- and micro-gametocyte (gamogony) or undergoes asexual division (merogony), resulting in 2–4 piroplasms being released when the erythrocyte ruptures. Repeating rounds of merogony can occur indefinitely while the felid is alive. When a vector-competent tick attaches to feed on an infected felid, it ingests the piroplasms along with their blood meal. The gametocyte piroplasms will then undergo sexual development (amphimixis) within the gut of the tick to form, first a zygote, then kinetes before migrating and encysting in the tick’s salivary glands, where they undergo additional replication (sporogony). Once the tick molts into a nymph or adult, the encysted sporozoites are ready to be released in the saliva of the feeding tick. Thus, the cycle begins anew. A detailed description of each *C. felis* life cycle stage is provided below and is based on what is known of *Theileria* spp., a close relative with a presumed similar cycle (Figure 3).

### 5.2. Asexual Schizogony

**Figure 4:**
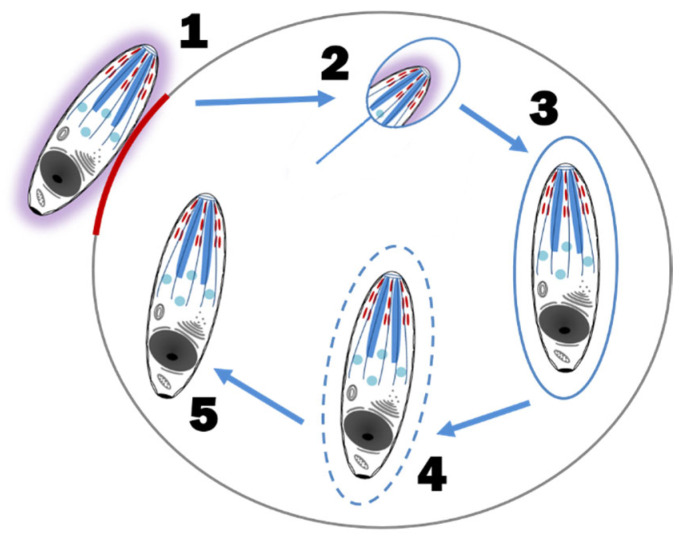
Sporozoite attachment and entry into a host cell. (1) A ‘slug trail’ of adhesive proteins (red) released from the micronemes of the apical complex allow actomyosin molecules of the sporozoite cell membrane to attach to the host cell resulting in a gliding motion, (2) sporozoite internalization via host cell membrane zippering with concurrent loss of its fibrillar coat (purple), (3) sporozoite withing the host cell membrane-bound vacuole, (4) host membrane separated and dissolved by the sporozoite, and (5) sporozoite free within the host cytoplasm coopts the host cell microtubule network for replication.

**Figure 5:**
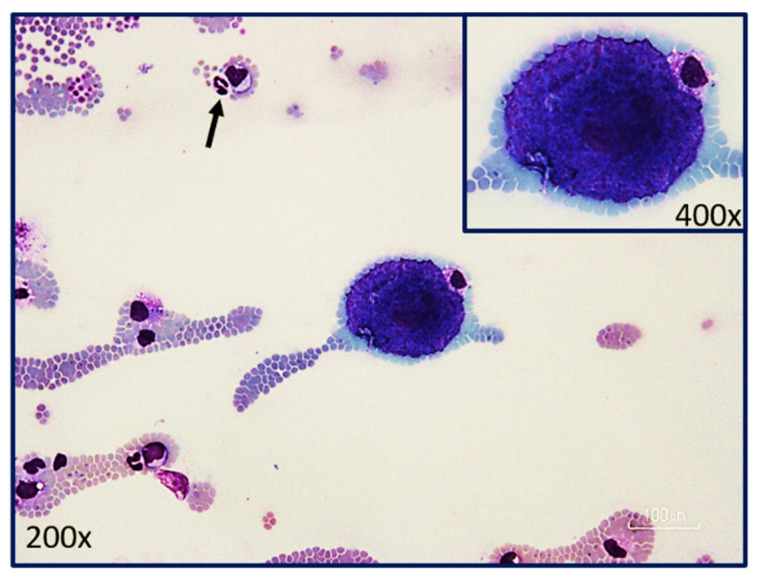
Merozoite-laden schizont or Koch’s body (insert) at the feathered edge of a blood smear from a cat with acute cytauxzoonosis. Note: neutrophil of 12–14 microns in diameter (black arrow) allows sizing of this schizont (54–63 microns) approximately 4.5 times larger than the neutrophil. (Photograph provided by Yvonne Wikander, DVM, MS, DACVP).

The rapid multiplication of *C. felis* within a monocyte is called schizogony and the merozoite-laden monocytes are called schizonts or Koch’s bodies [4]. Asexual schizogony begins after sporozoite-laden tick saliva is released into the feeding lesion around an embedded tick’s mouthparts [4,12]. The immotile sporozoites, which have a fuzzy coat made up of fibrillar material and hypervariable surface proteins, rely on random contact with monocytes to begin their attachment and internalization. Unlike some apicomplexans, *Theileria* spp. and presumably *C. felis*, does not require an apical-end orientation with the host cell. It can enter the host cell in any orientation and does so within three minutes of contact. The process of invasion is as follows; (1) the organism recognizes and attaches to the host membrane, (2) using gliding motility, a junction is formed between the parasite and the host cell, (3) as the parasite internalizes via host membrane zippering, it’s fuzzy coat is shed, (4) the host membrane surrounding the parasite is separated and dissolved, leaving the organism free within the host cytoplasm, and (5) the parasite takes control of the host cell’s microtubular network for its own development [4,16] (Figure 4). The mechanism by which the parasite hijacks the host cell to multiply and/or evade the host’s immune system is unknown [19]. In addition, it is unknown if *C. felis* blocks monocyte apoptosis as *Theileria parva* blocks lymphocyte apoptosis. Regardless, once established within a host monocyte, ultrastructural changes to the parasite’s organelles and outer membrane and sequential fissions results in a multilobulated, multinucleated mass connected by cytoplasmic bridges [4,20]. Each lobe has a nucleus, a mitochondrion, and related organelles [20]. Eventually the cytoplasmic bridges separate, leaving multiple mature intracytoplasmic uninuclear merozoites [4,20]. Mature schizonts (measuring 25–60 μm in diameter) rupture, releasing merozoites into the blood [21] (Figure 5). As with all extracellular phases of *C. felis* development, merozoites form a fuzzy coat of fibrillar material to assist in its invasion of the next host cell, the erythrocyte [4,20].

### 5.3. Asexual Merogony

**Figure 6:**
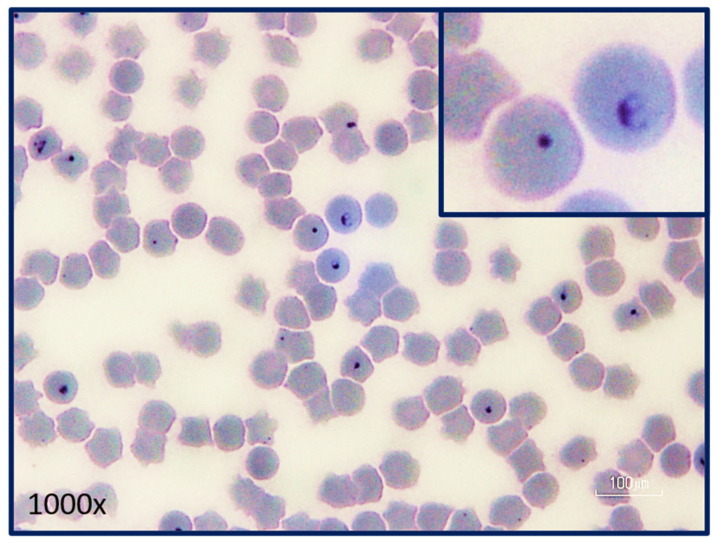
Intra-erythrocytic signet ring piroplasms in a blood smear from a cat with acute cytauxzoonosis. Note the pair of merozoites in one of the erythrocytes demonstrating active merogony (inset). The remaining intra-erythrocytic piroplasms could be trophozoites, merozoites, or gametocytes which cannot be differentiated via light microscopy. (Photograph provided by Yvonne Wikander, DVM, MS, DACVP).

The budding fission of *C. felis* within erythrocytes is called merogony and occurs when a trophozoite divides into a pair or tetrad of merozoites [4]. Merozoites enter erythrocytes in the same fashion that sporozoites enter monocytes [4,20]. The internalized parasite develops into a trophozoite, phagocytoses or pinocytosis the host cytoplasm through their pellicular micropores, and then asexually divides into 2–4 merozoites. Erythrocyte rupture releases the merozoites into the host blood to begin the asexual merogony cycle again. With that said, not all trophozoites produce merozoites. Some develop into haploid gametocytes (gamogony), which do not reproduce within erythrocytes [4]. Gametocytes are larger than merozoites and have unusual shapes that are not visible via light microscopy. As such, trophozoites, merozoites, and gametocytes all appear as 1–2 μm signet ring piroplasms within erythrocytes, microscopically (Figure 6).

### 5.4. Sexual Reproduction

Amphimixis, the fusion of two gametes of *Theileria* spp. and, presumably *C. felis*, occurs within the gut of the tick vector [4]. Ticks coat their food bolus with organized chitin microfibrils held together with specific proteins (peritrophins) within a thick proteoglycan matrix. This porous coating, called the peritrophic matrix, acts as a barrier protecting the tick’s gut epithelium from infectious organisms as well as mechanical and chemical damage, while improving the efficiency of digestion [22,23,24]. Intra-erythrocyte merozoites, trophozoites, and gametocytes enter the tick gut lumen along with the bloodmeal coated with the previously described peritrophic matrix [4]. Within five days of ingestion, and as the erythrocytes are lysed, the merozoites and trophozoites are destroyed and digested, whereas the gametocytes reorganize their microtubules and cytoplasm forming haploid anisogametes [4]. Macrogametes (female form) take on a rounded shape, whereas microgametes (male form) stretch out and form a ray body, which has the shape of an arrowhead with trailing arms [4]. When a micro- and macrogamete come into close contact, they attach to each other via small fibrils, a small tube forms from one gamete to the other, the nucleus of the microgamete fuses with the nucleus of the macrogamete, and a motile diploid zygote is formed [4]. The arrowhead of the zygote releases chitinases and proteinases to dissolve a path through the peritrophic matrix, allowing its escape into the ecto-peritrophic space next to the tick’s intestinal epithelial cells [4]. The zygote immediately enters an intestinal epithelial cell via the same mechanisms previously described [4]. Once inside the host cell’s cytoplasm, the zygote spheres and its arrowhead disappears, and multiple motile haploid kinetes are formed via meiosis [4]. The kinetes then exit the cell and enter the tick’s hemolymph, an open circulatory system equivalent to mammalian blood [4]. This process occurs within 13–34 days of tick ingestion [4].

### 5.5. Asexual Sporogony

Sporogony is the production of infective sporozoites. This stage starts with the kinetes travelling to the tick’s salivary acini and gaining entry in the same way they entered monocytes, erythrocytes, and tick intestinal epithelial cells [4]. The intracellular kinete then forms a sporont, a large multinucleated syncytial cell that further develops into a sporoblast, a sporont with a three-dimensional branching network. During this process, the tick’s salivary acinar cell hypertrophies to accommodate the enlarging sporoblast. At this point the tick undergoes ecdysis (molting of its cuticle) and the sporoblast remains dormant. In this way, the *C. felis* organisms remain within their tick host from one life stage to the next, also called transstadial or horizontal transmission. Within 48 h of tick attachment to a host, the dormant sporoblast matures, apical complexes are formed, and sporozoites ‘bud’ off via multiple fission. These sporozoites are released into the tick saliva and inoculated into the host. If the host is a felid, the life cycle is repeated. Sporogony is an asynchronous event with various stages of sporozoite development occurring at the same time within salivary acinar cells. The result is a nearly continuous production, and release, of sporozoites into the tick’s saliva and thus, the felid host.

## 6. Transmission Vectors & Definitive Host

Thus far, two ixodid ticks have been identified as competent biological vectors of *C. felis* in the U.S., *Amblyomma americanum* and *Dermacentor variabilis* [25,26,27,28]. Like all ixodid ticks, they have a life cycle with four stages, each of which can survive several months waiting for a host: (1) egg, (2) larva, (3) nymph, and (4) adult, with the latter three stages requiring a bloodmeal prior to transitioning to the next stage [29,30]. Depending on environmental conditions, mainly temperature and humidity, the life cycle of these ticks can take 2–3 years to complete [29]. To identify a host, ticks engage in a behavior called ‘questing’ which includes climbing vegetation to an appropriate height, extending its two rostral legs for host attachment, and responding to several potential factors, e.g., movement, carbon dioxide, size, color, odor, touch, and sweat [29,30]. Once piggy-backed onto its host, the tick can take up to several hours to find an appropriate feeding site [30]. To obtain a bloodmeal, the tick uses toothed chelicerae to cut through the dermis and allow for the barbed hypostome to penetrate the skin. The mouthparts secrete a cement or latex-like material to hold the hypostome in place, during the several days of feeding [29,30]. During feeding, the tick releases many substances in its saliva, including anti-coagulants, anti-inflammatories, analgesics, and pathogens, like *C. felis* [31,32]. These salivary substances mitigate the host immune responses likely assisting pathogens like *C. felis* to become established. In addition, the salivary composition changes over time to ensure an uninterrupted bloodmeal and reaches peak volumes during the final 24–48 h of attachment. It is unknown how changes in saliva composition affects *C. felis* sporozoite transmission to the felid host, if at all. Regardless of life stage, once feeding is complete, the tick falls off, digests its bloodmeal, and molts into the next life stage in preparation to overwinter or lay an egg mass of several thousand eggs [30]. The larval, nymph, and adult forms of vector competent ticks may become infected with *C. felis* during acquisition of a bloodmeal from a piroplasm-carrying felid. After molting into the nymph or adult forms, these ticks transmit *C. felis* to their next felid host. Although both tick vectors have similar life cycles and a preference for geographic areas with ground debris, long grasses, and brushy to wooded areas, they also have important differences [29,33].

### 6.1. Amblyomma americanum

*Amblyomma americanum*, the Lone star tick, is indigenous to the southeastern and mid-central U.S. with a range that overlaps that of *D. variabilis*, and is expanding west and north with climate changes [29,34]. It is a brown sexually dimorphic species in which the females demonstrate a white spot on their central caudal scutum, while the males demonstrate small white spots along the margin of their scutum [29]. This is an aggressive, indiscriminate tick species in which all life stages will feed on any size animal, including cats. Adults are most active in early spring to mid-summer, nymphs in late spring to early fall, and larvae in late summer to early fall [29]. However, activity will vary with region and location. A nationwide tick infestation study found that *A. americanum* ticks were the second most common tick found on cats, most being in larval forms (39.1%) with fewer adults and nymphs [35]. *Amblyomma americanum* were generally found on the ventral regions of cats, especially the tail and perianal regions [36]. Although feline tick infestations peak in mid-summer, studies have identified cats with ticks all year around, including exclusively indoor cats [35,36]. As such, *C. felis* infections could occur in any season and in any cat regardless of their lifestyle.

Two studies have demonstrated *A. americanum* is a competent vector for *C. felis* transmission. The first study used *A. americanum* adults that had been fed to repletion as nymphs, on a chronically *C. felis*-infected cat with 0.9% *C. felis* parasitemia, to infect four *C. felis* naïve cats [25]. All four *C. felis* naïve cats exhibited typical cytauxzoonosis signs with evidence of infection observed in blood and tissue evaluation. Another study used *A. americanum* nymphs that had been fed to repletion as larvae, on a chronically *C. felis*-infected cat with 0.004% *C. felis* parasitemia, to infect three *C. felis* naïve cats [27]. All three *C. felis* naïve cats exhibited typical cytauxzoonosis signs with evidence of infection on blood and tissue evaluation. Transmission studies have determined *C. felis* sporozoite transfer can occur within 36–72 h of feeding initiation by a tick of this species [26,28].

### 6.2. Dermacentor variabilis

*Dermacentor variabilis*, the American dog tick, has a large geographic distribution extending over the entire eastern half of the U.S., with a focal region on the west coast [31,37,38]. It is a brown tick with gold or silver markings on its scutum. Larvae of this species prefer to feed on rodent-sized hosts and are most active in early spring through mid-summer, nymphs prefer opossum-sized hosts and are most active in early summer to early fall, and adult *D. variabilis* ticks feed most commonly on dog to deer-sized mammalian hosts, with their greatest activity in early spring to early fall. A recent, nationwide tick infestation study found that *D. variabilis* ticks were the third most common tick species found on cats, most being adult females (59.2%), with far fewer larvae and rare nymphs recovered [35]. *Dermacentor variabilis* ticks were generally found in the dorsal regions of cats, especially the head and ears [36].

Two studies have demonstrated that *D. variabilis* is a competent vector for *C. felis* transmission. In the first study, *D. variabilis* nymphs were allowed to feed to repletion on a splenectomized chronically *C. felis*-infected wild-caught bobcat with 40% *C. felis* parasitemia [39]. Once the ticks molted into adults, they were allowed to feed on a splenectomized cat, resulting in typical acute cytauxzoonosis signs and findings. Another study used laboratory-reared *D. variabilis* nymphs allowed to feed to repletion on *C. felis*-infected cats [20]. After molting into adults, they were allowed to feed on *C. felis* naïve cats. As with the previous experiment, the *C. felis* naïve cats also demonstrated typical acute cytauxzoonosis signs and findings. To this author’s knowledge, transmission time studies have not been performed to determine the transfer time of *C. felis* to felids by *D. variabilis*.

Although the above studies from 1984 [39] and 1992 [20] demonstrated that *D. variabilis* is capable of transmitting *C. felis*, three studies from 2009 to 2019 compared both the *A. americanum* and *D. variabilis* competency to transmit *C. felis* to domestic cats and found it was only transmitted by *A. americanum* [25,27,40]. The reason for transmission failure in these recent studies is unknown. Perhaps some *D. variabilis* lineages are refractory to *C. felis*. Additionally, since adult *D. variabilis* ticks more commonly feed on cats than juvenile stages, it makes sense that transmission by *D. variabilis* is less common. This contrasts with *A. americanum*, for which larval, nymphal and adult life stages will feed on cats providing multiple opportunities for *C. felis* acquisition and transmission. 

Given their expanding distribution, aggressive nature, observation frequency on feline hosts, and correlation of geographic distribution with clinical cases of cytauxzoonosis, *A. americanum* is likely the more significant vector for *C. felis* transmission to felids, especially domestic felids. As the geographic ranges of *C. felis* vector competent ticks continue to expand, it is likely that the number of cytauxzoonosis cases seen in felid intermediate hosts will also increase.

## 7. Felid Intermediate Hosts

Members of the family Felidae serve as intermediate hosts of *C. felis* and infection reservoirs. Examples of felids naturally infected with *C. felis* in the U.S. include bobcats (*Lynx rufus*) [41,42,43,44,45], domestic cats (*Felis catus*) [46,47], cougars (*Puma concolor*) [48,49,50], captive lions (*Panthera leo*), and tigers (*Panthera tigris*) [51,52]. Of these, only the bobcat and domestic cat have been confirmed as competent reservoirs for *C. felis* transmission to other felids via vector competent tick species [25,39,40,53].

For many decades, the bobcat was assumed to be the primary reservoir for *C. felis* in the U.S. Studies demonstrated that most bobcats show few clinical signs with a shortened schizont phase [41,42,43]. One prevalence study that evaluated the distribution and prevalence of *C. felis* carriers in wild felids (*n* = 705) using nested PCR and sequence analysis, found that 138 of 696 bobcats over 14 states tested positive for *C. felis* [45]. The individual state prevalence of *C. felis* in bobcats varied from 0 to 79%, with a strong association between the *C. felis* prevalence and the distribution of *A. americanum*. With that said, there are also reports of acute cases in bobcats leading to death [42,54]. It is possible that acute cases of this disease occur more frequently than thought in bobcats but are never seen due to the hidden life of these small predators.

**Figure 7:**
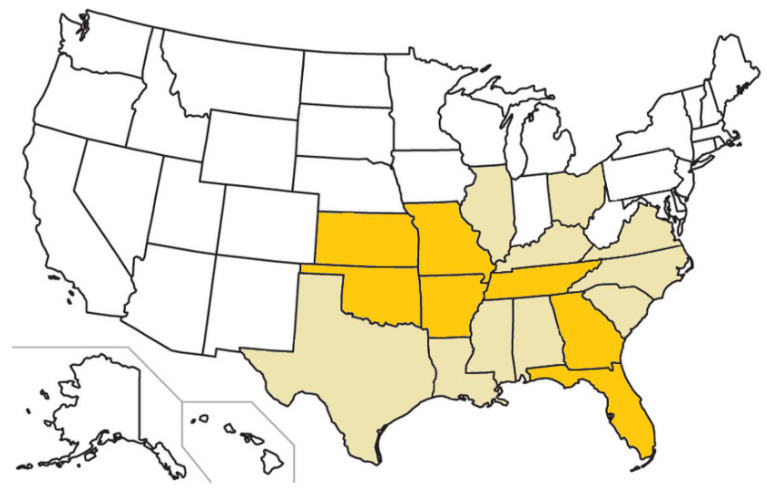
U.S. states with confirmed feline cytauxzoonosis cases (beige and orange) and states in which carrier cat populations have been identified (orange).

At one time, domestic cats were considered a dead-end host for *C. felis* as infection commonly ended in death [2,3,55,56]. However, as more studies explored *C. felis* prevalence among domestic cats, this assumption came under scrutiny [2,28,40,53,57,58,59,60,61] (Figure 7). Four studies between 2007 and 2020 in the U.S. looked at the prevalence of *C. felis* carrier domestic cats using molecular diagnostic assays. One study evaluated 961 trap-neuter-release cats in Florida (*n* = 494), North Carolina (*n* = 392), and Tennessee (*n* = 75) identifying only 3 positive cases (0.3% overall prevalence) [59]. Another study identified 56 positive cases out of 902 healthy client-owned cats in Arkansas (25/161 cats; 15.5% prevalence), Missouri (8/62 cats; 12.9% prevalence), and Oklahoma (23/679 cats; 3.4% prevalence) [53]. A third study identified 3 positives out of 672 healthy free-roaming (presumed trap-and-release) cats in Oklahoma (3/380 cats; 0.8% prevalence) and Iowa (0/292 cats: 0% prevalence) [28]. The last study evaluated 1,104 feral (*n* = 216), owned (*n* = 351), and rescued (*n* = 537) domestic cats with no known history of *Cytauxzoon* infection in eastern Kansas, identifying 270 positive cases (25.8% overall prevalence) [5]. These studies suggest that domestic cats can, and are likely to act, as significant infection reservoirs in these areas. In *A. americanum* endemic areas especially, both feral and owned domestic cats that survive acute cytauxzoonosis can serve as infection reservoirs for indigenous Lone star ticks to perpetuate the *C. felis* life cycle.

## 8. Cytauxzoonosis

Cytauxzoonosis can present as either an acute life-threatening form or as a subclinical form, with the subclinical form generally diagnosed incidentally. The acute form is clinically evident during the leukocyte (schizogenous) phase of *C. felis* infection and is most typically seen in late spring with fewer cases seen in early fall, corresponding with tick vector activity [21,62]. The mortality rate for acutely infected cats presented to veterinary facilities is very high (40–100%) depending on whether appropriate and timely treatment is initiated or not. The chronic subclinical form is the result of surviving an acute infectious form and is evident during the erythrocyte (merogonous) phase of *C. felis* infection. Felids with the subclinical form act as a reservoir for future tick and felid infections and do not benefit from any known treatments.

### 8.1. Acute Disease

Cats with acute cytauxzoonosis commonly present to the veterinary clinic for acute lethargy, anorexia, depression, and fever approximately 11 days post infection (dpi) [2,40,55,57,58]. On physical exam, these cats are dehydrated, pyrexic (103–106 °F; 39.4–41.1 °C), have pale mucous membranes, and splenomegaly [2,40,55,57,58,63]. As the disease progresses, pyrexia resolves and drops to subnormal with icterus and dyspnea developing shortly thereafter (16–21 dpi) [3,40,58,62]. Complete blood count and serum biochemistry changes are seen at around 13 dpi. Cytopenias are variable and can include: (1) marked non-regenerative anemia (0.10–0.18 L/L; RI 0.29–0.48 L/L), (2) leukopenia (1.3–6.5 × 10^9^/L; RI 5.4–2.3 × 10^9^/L), (3) neutrophilia (10.45–22.09 × 10^9^/L; RI 2.5–8.5 × 10^9^/L), (4) lymphocytosis (21.2–31.63 × 10^9^/L; RI 1.2–8.0 × 10^9^/L), and (5) moderate to marked thrombocytopenia (12.9–73.3 × 10^9^/L; RI 300–800 × 10^9^/L) [40,55,63]. Serum biochemistry and urinalysis findings commonly include decreased albumin, increased glucose (7.94–12.15 mmol/L; RI 3.50–8.32 mmol/L), increased Alanine Aminotransferase (ALT), increased total bilirubin (15.39–141.93 mmol/L; RI 0.0–8.55 mmol/L), and bilirubinuria. When blood smears are evaluated, schizont-laden monocytes may or may not be seen at the feathered edge (Figure 5), but 1–2 μm signet ring piroplasms within erythrocytes will be seen by 18 dpi [40] (Figure 6). Blood samples submitted for real-time PCR will test positive for *C. felis* at around 17 dpi. More sensitive PCR testing, e.g., droplet digital PCR (ddPCR), could improve early diagnosis of acute disease allowing for earlier institution of life-saving treatment [64].

Death, generally occurring at about 21 dpi, has long been assumed to be due to vascular obstruction by engorged schizonts, resulting in multiorgan failure due to hypoxic injury. However, recent studies have suggested that along with hypoxic injury, local and systemic immune responses to proinflammatory substances released by neutrophils and schizogenous monocytes are responsible for much of the morbidity and mortality seen in cytauxzoonosis [21,65,66,67]. Leukocyte activation results in proinflammatory cytokine release which affect platelets and endothelial cells resulting in a hypercoagulable state that may culminate in disseminated intravascular coagulation (DIC) [67,68]. Studies have measured a significantly higher systemic concentration of the proinflammatory cytokine TNF-α in cats that eventually died of the disease versus those that survived [65,66]. In addition, CD18, an adhesion molecule that likely attaches infected monocytes to activated endothelium, was also upregulated in cats that died versus those that recovered [65]. It is not yet known whether the immune dysregulation seen is caused by the parasites themselves or by the secondary responses to proinflammatory cytokines. Cats that succumbed to cytauxzoonosis had typical interstitial pneumonia findings on lung histopathology that included thickened pulmonary interstitium due to edema and neutrophilic infiltrates, neutrophilic alveolar exudate, and evidence of vasculitis. Immunohistochemistry demonstrated “a significant, widespread, qualitative increase in the expression of the pro-inflammatory cytokines TNF-α, IL-1β, IL-6, as well as inducible nitric oxide synthase (iNOS)”, while uninfected cat tissues demonstrated no or minimal expression of iNOS [66]. Expression of iNOS is commonly found in human and animals with acute pulmonary distress syndrome [66]. In the aforementioned studies, schizonts stained positive for iNOS, suggesting they were producing reactive nitrogen intermediates [66]. These intermediates can then activate TNF-α, IL-1β, and/or IL-6. TNF-α and IL-1β upregulate CD18, the adhesion molecule previously mentioned, while TNF-α activates endothelial cells and induces them to express MHC-II [65,66]. Activated endothelial cells contract, causing local edema, and release nitrous oxide (NO), which contributes to local tissue inflammation and damage, and results in hypotensive shock and microvascular damage [66].

**Figure 8:**
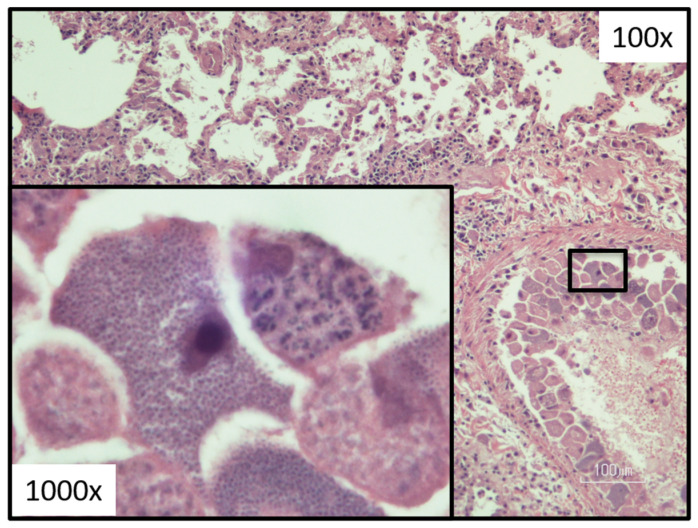
Lung tissue histology demonstrating a vessel with many schizonts (merozoite-laden monocytes in varies stages of development). (Photograph provided by Yvonne Wikander, DVM, MS, DACVP).

On necropsy, felids that have succumbed to acute cytauxzoonosis demonstrate generalized icterus, splenomegaly, lymphadenopathy, petechia and ecchymosis on the heart and lungs, clear yellow serous pericardial effusion, interstitial pneumonia, and large numbers of schizonts within the vasculature attached to endothelial cells of most organs (Figure 8); with the liver, lung, spleen, and lymph nodes most effected [2,21,58,63]. When examined, the brain demonstrates schizogenous vascular occlusion with secondary ischemia and necrosis of neurons as well [58,69,70]. The intravascular mega-schizonts are a prominent diagnostic feature in these cases and typically measure 25–60 μm in diameter, but may reach 250 μm in diameter with an enlarged nucleus, prominent nucleolus, and cytoplasm full of variably mature basophilic merozoites [21]. The cause of death in acute *Cytauxzoon* cases is a combination of hypoxic injury and local and systemic immune responses to proinflammatory cytokines leading to poor pulmonary ventilation, decreased gas exchange, and/or a procoagulant state resulting in DIC. Additional studies are needed to determine how the host immune response to this pathogen differs during schizogenous and erythrocytic parasite phases, what factors mitigate those host immune responses, and how we might manipulate the feline immune response to improve survival of these patients.

### 8.2. Subclinical Disease

Felids that survive the acute phase of this disease, show evidence of erythrocyte regeneration at 18–22 dpi, with resolution of subnormal body temperature and clinical signs shortly thereafter (23–24 dpi) [40]. Normalization of erythrocyte indices is complete by 43 dpi. These survivors manifest the chronic subclinical form of this disease by entering a persistently parasitized erythrocyte stage [59,63]. It is unknown if the intra-erythrocytic piroplasms are predominantly trophozoites, merozoites, or gametocytes, or if the distinction matters. Felids with chronic disease are completely asymptomatic and remain persistently infected for years, if not for life [57]. They appear to be protected from re-developing clinical disease with additional *C. felis* challenges, provided they survived a previous schizogenous phase. However, this reprieve may not be lifelong or may not be equally protective against heterologous strains of the pathogen. A study evaluating the prevalence of *C. felis* in southern Illinois wild-caught bobcats, identified one individual that appeared to have been infected with a different strain (based on Internal Transcribed Spacer 1 (ITS-1) single nucleotide polymorphism) of *C. felis* between captures [44]. More recently, a domestic shorthair cat successfully treated for acute cytauxzoonosis seven years prior, was presented with a repeat *C. felis* infection confirmed on splenic histopathology [71]. Because survival of the schizogenous phase is required for future protection, cats transfused with piroplasm-laden blood will harbor piroplasms as a chronic carrier but are not immune to *C. felis* challenge [41,72]. Due to their asymptomatic nature, carrier cats are typically only diagnosed with *C. felis* if (1) they have a known history of surviving acute cytauxzoonosis, (2) piroplasms are identified on a blood smear exam, and/or (3) they test positive for *C. felis* via PCR. Further studies are needed to determine if *C. felis* reservoir cats are more likely to be infected with certain *C. felis* strains, have co-infections with other organisms and, if so, how that might affect the cat’s carrier status.

## 9. Diagnosis

The differential diagnosis for the acute cytauxzoonosis clinical signs of fever, lethargy, icterus, dyspnea, and anemia could include cholangiohepatitis, hepatic lipidosis, pancreatitis, triaditis, sepsis, immune-mediated hemolytic anemia, oxidative damaging toxins (e.g., acetaminophen, *Allium* spp), neoplasia, tularemia, feline infectious peritonitis, and hemotropic mycoplasma, to name a few. Since no rapid in-clinic test is available to diagnose acute cytauxzoonosis, it is generally diagnosed by schizont (Figure 5) and/or intra-erythrocytic signet ring (Figure 6) identification at an antemortem blood smear review, or schizont identification on postmortem tissue sample histopathology (Figure 8). Although blood smear review and tissue histopathology are considered equally diagnostic for this disease [5], a recent paper concluded that ‘novice observers’ were better able to identify schizonts in splenic fine needle biopsy samples (77.1% sensitivity; 94.4% specificity) than lymph node aspirates (52.8% sensitivity; 96.4% specificity) or blood smears (41.7% sensitivity; 96.9% specificity) [73]. When a blood smear demonstrates signet ring (aka. piroplasm) erythroid hemoparasites in a cat, the three main differentials include: (1) *Mycoplasma hemofelis*, (2) *Cytauxzoon felis*, and (3) *Babesia* spp. *Mycoplasma hemofelis*, one of the most common feline hemoparasites, is generally associated with a strongly regenerative hemolytic anemia, and coccoid or signet ring shaped organisms located on the erythrocyte membrane and/or in the background [74]. *Cytauxzoon felis* infections are often associated with a variable non-regenerative anemia, signet ring shaped organisms within erythrocytes and rarely in the background, and/or schizonts at the blood smear feathered edge or in tissue sample histology. Although immunocompetent cats have been found to carry several *Babesia* species without ill effects in Europe, Asia, the Middle East, and the Americas, four *Babesia* species in South Africa including *Babesia leo*, *Babesia lengau*, and *Babesia* spp. Western Cape, and *Babesia felis* are associated with disease which can include a regenerative anemia and intra-erythrocytic signet rings shaped organisms often arranged in tetrads [75]. Although none of the latter four *Babesia* spp. have been reported in the U.S., it should be a differential for any symptomatic felid with a history of travel to Africa, especially the southern coastal regions. PCR is more readily available to identify *M. hemofelis* and/or *Babesia* spp., than for *C. felis*. That said, PCR testing for *C. felis* is now available through the Vector-borne Disease Diagnostic Laboratory (North Carolina State University College of Veterinary Medicine), Zoologix Inc. (California), and Bioingentech Ltd. (Chile). Additionally, a probe-based ddPCR assay has been proposed for early identification (24 h prior to clinical signs) of cytauxzoonosis as well as for evidence of treatment response [64]. Although not currently commercially available, ddPCR may augment or replace other PCR techniques in the future. Since subacute and early acute infections may not present with visible intra-erythrocytic signet rings and/or schizonts in *C. felis* infected cats, an in-hospital test needs to be developed for rapid diagnoses of acute cytauxzoonosis cases to initiate potentially lifesaving treatment for these cats. With that said, a diagnosis of acute cytauxzoonosis in any cat living in or near *C. felis* endemic areas with typical clinical signs can be made by visualization of either schizonts and/or intra-erythrocytic signet rings via histopathology or blood smear samples. Since each of the previously mentioned piroplasm diseases are treated differently, proper identification is critical for successful outcomes in feline patients. Thus, in locations or regions where these feline piroplasm diseases overlap, DNA sequencing may be indicated for proper species identification.

## 10. Treatment Options

All treatments for acute *C. felis* infections have incorporated basic supportive care methods including intravenous fluids for dehydration and hypotension, antimicrobials to treat concurrent septicemia, and heparin to prevent thrombus formation and DIC. Several targeted anti-protozoal treatments have been proposed and attempted for *C. felis* with variable success. Parvaquone and buparvaquone, used to treat *Theileria parva* in cattle, were investigated, but found to be ineffective in treating experimentally induced cytauxzoonosis cases [72,76]. Imidocarb dipropionate, a urea derivative used to treat *Babesia canis*, demonstrated limited, inconsistent results, and was also abandoned as a treatment candidate [57,72,77]. Dimethazine aceturate, used to treat trypanosomiasis in livestock, was equally ineffective in the treatment of naturally occurring acute and chronic *C. felis* infections in cats [47,78].

Currently, the most effective treatment is a combination of atovaquone (15 mg/kg PO q 8 h × 10 days) and azithromycin (10 mg/kg PO q 24 h × 10 days) which provides a 60% success rate in treated cats, if initiated in a timely manner [79]. Atovaquone, a malaria treatment candidate, is a ubiquinone (Coenzyme Q) analogue that targets the cytochrome b subunit of the mitochondrial electron transport chain [18,80,81,82]. Early studies of atovaquone as a potential anti-malarial medication, demonstrated that parasite mitochondrial membrane potential collapsed within minutes of treatment, while leaving the host mammalian mitochondria unaffected [83]. Subsequent studies explored energy metabolism in *Plasmodium* spp. and found their mitochondrial energy production in the erythrocytic stage was very slow, possibly explaining the poor *Plasmodium* response to atovaquone [84]. As stated previously, Apicomplexans appear to be missing at least two of the necessary mitochondrial ETC subunits needed to perform oxidative phosphorylation [18]. As such, atovaquone may not inhibit a vital function of their mitochondrion resulting in a less than optimal response to this drug. Azithromycin, a macrolide antibiotic, inhibits protein translation at the parasite mitochondrial ribosome level and, thus, the parasite’s growth [18,80,81,82]. With the best treatment resulting in 40% mortality, improved treatment protocols are needed. Many factors may influence patient response to treatment including the timing of treatment initiation, the patient’s immune response to the parasite, the sporozoite load injected, and parasite strain virulence to name a few [57,85]. Although further investigation is needed to assess each of these factors and their contribution to the disease process and patient response, no *C. felis* strain banks nor in vitro experimental systems exist to facilitate the needed research. Thus far, studies trying to identify different pathogenic or virulent strains using the ITS region of the 18S rRNA gene have determined there is no genotype association with geography or clinical outcome of this disease [46,86]. Additionally, no treatment protocols are available to clear infection of persistently infected felids to reduce infection reservoir potential.

## 11. Control & Prevention

Since cats surviving the acute schizogenous phase appear to have protection from re-developing clinical signs with future infections for an undetermined duration, it is assumed they form a protective immune response [41,72,87]. As such, a vaccine could potentially be developed if an appropriate protein candidate were found. To that end, Tarigo et al. sequenced the *C. felis* genome and identified 4300 potential protein-coding genes [85]. To date, two potential vaccine candidates have been examined. The highly conserved Cf76 gene, like the *T. parva* p67 gene, encodes a protein expressed during the schizont phase and is recognized by the feline humoral immune system and the AMA-1 gene, encoding an apical membrane antigen [2,87,88]. However, to date, no vaccine has been developed. To produce a successful vaccine, more research is needed to identify and confirm conserved, immunogenic, and protective antigens. In addition, more studies are needed to better understand genetic determinants of virulence, transmission, and treatment success, especially in the context of *C. felis* strain heterogeneity.

Since no *C. felis* vaccine exists and there is no consistently effective treatment, control of this disease is based on transmission prevention by eliminating, or limiting, exposure to the tick vectors. It is not uncommon for multi-cat households to see multiple cases of cytauxzoonosis. As such, limiting cats’ outdoor time during peak tick season, manual tick removal, and/or treatment with acaricides remain the mainstay of control for this disease [6,53,86,87]. Two acaricides have demonstrated good to excellent efficacy in preventing *C. felis* transmission by *A. americanum*, under experimental conditions. The first product, imidacloprid 10%/flumethrin 4.5% collar (Seresto, Elanco Inc, Greenfield, IN, USA), demonstrated 100% efficacy in preventing adult *A. americanum* attachment and feeding, and thus transmission of *C. felis*, in 10 cats [89]. Flumethrin, a synthetic pyrethroid, affects the neuronal sodium channels of mites and ticks resulting in their death as well as acting as a repellant. Although cats are generally sensitive to pyrethroids, this synthetic form does not require hepatic glucuronidation, rendering it safe for use in cats. The second product, selamectin/sarolaner (Revolution Plus, Zoetis Inc., Parsippany, NJ, USA), demonstrated >90% efficacy in reducing *A. americanum* and *D. variabilis* tick counts 72 h after infestation and preventing transmission of *C. felis* in 8 cats, when applied monthly [90]. Sarolaner, the isoxazoline compound in this product, causes paralysis and death of ticks via neural blockade. Since *C. felis* can be transmitted from the tick to its felid host within as little as 36 h, it is critical to use preventative products that either repel or kill ticks prior to parasite transmission. Other current or future products that kill *A. americanum* and/or *D. variabilis* within 36 h should be equally efficacious in *C. felis* transmission reduction. Additional research is needed to improve preventative treatments, including considering developing a molecule that would block earlier parasite developmental stages (e.g., sporozoite development in the tick), or development of a vaccine against the tick vector.

## 12. Summary & Future Considerations

Cytauxzoonosis is caused by *Cytauxzoon felis*, a protozoal apicomplexan hemoparasite of felids in the *Theileria* & *Cytauxzoon* clade of Piroplasmidae, endemic to North America. In the last four decades, much has been elucidated about this organism’s complex life cycle, transmission, and the disease it causes in felids. Although it appears that most, if not all, felids can become infected, the known competent hosts include bobcats (*L. rufus*) and domestic cats (*F. catus*). Once within a host, *C. felis* begins the schizogenous phase of asexual replication, which can cause severe illness and death in its host due to proinflammatory cytokine stimulated damage, DIC secondary to endothelial damage, hypoxic injury of multiple organs, and interstitial pneumonia with poor gas exchange exacerbating hypoxemia. If the host animal survives the schizogenous phase, the *C. felis* organism enters a perpetual erythrocytic phase causing the asymptomatic host to remain persistently parasitemic, acting as a disease reservoir for years. Although clinical disease upon subsequent re-infection is uncommon, it appears that infection with a different *C. felis* strain can lead to additional acute life-threatening schizogenous phases in the same host. The cornerstone of *C. felis* prevention is avoidance of transmission via tick control. As such, all cats living in endemic areas, including indoor exclusive cats and those with subclinical infection, should be treated year-round with acaricide products that are known to repel or kill ticks rapidly.

A vaccine and more effective, affordable treatments are desperately needed, as is an affordable rapid diagnostic test to confirm diagnosis early in the disease process and to identify *C. felis* carrier cats. In addition, more studies are needed to better understand factors affecting infection, disease, and treatment. This includes the minimum sporozoite load necessary for infection, whether sporozoite load affects the length and severity of the schizogenous phase, determining the host immune responses to the parasite and what mitigates it, identifying *C. felis* virulence factors, developing *C. felis* strain banks, and determining how treatment timing effects outcome, to mention a few. Research of this caliber would require significant funds; however, funding opportunities for researching this organism (especially basic research) are currently limited. Additionally, there is no in vitro experimental system available for *C. felis*, so investigating many of these questions requires the use of deliberately infected cats, raising ethical concerns. As such, much of the current knowledge about *C. felis* assumes that it is similar to related Piroplasmidae species. These assumptions may be partially, or wholly, inaccurate. A good starting point would be to investigate: (1) the risk factors associated with acute cytauxzoonosis infection, (2) whether the incidence of acute cytauxzoonosis is changing over time, and (3) the prevalence of *C. felis* carrier cat populations.

## Data Availability

Not applicable.

## References

[1] Panait L.C., Mihalca A.D., Modry D., Jurankova J., Ionica A.M., Deak G., Gherman C.M., Heddergott M., Hodzic A., Veronesi F. (2021). Three new species of *Cytauxzoon* in European wild felids. Vet. Parasitol..

[2] Wang J.L., Li T.T., Liu G.H., Zhu X.Q., Yao C. (2017). Two Tales of *Cytauxzoon felis* Infections in Domestic Cats. Clin. Microbiol. Rev..

[3] Reichard M.E., Sanders T.L., Weerarathne P., Meinkoth J.H., Miller C.A., Scimeca R.C., Almazán C. (2021). Cytauxzoonosis in North America. Pathogens.

[4] Jalovecka M., Hajdusek O., Sojka D., Kopacek P., Malandrin L. (2018). The Complexity of Piroplasms Life Cycles. Front. Cell. Infect. Microbiol..

[5] Wikander Y., Anantatat T., Kang Q., Reif K.R. (2020). Prevalence of *Cytauxzoon felis* Infection-Carriers in Eastern Kansas Domestic Cats. Pathogens.

[6] Neitz W.O., Thomas A.D. (1948). *Cytauxzoon sylvicaprae* Gen. Nov., Spec. Nov., a Protozoon Responsible for a Hitherto Undescribed Disease in the Duiker, *Sylvicapra grimmia* (Linné). Onderstepoort J. Vet. Sci. Anim. Ind..

[7] Wagner J.E. (1976). A Fatal Cytauxzoonosis like Disease in Cats. J. Am. Vet. Med. Assoc..

[8] Ferris D.H. (1979). A Progress Report on the Status of a New Disease of American Cats: Cytauxzoonosis. Comp. Immunol. Microbiol. Infect. Dis..

[9] Nijhof A.M., Pillay V., Steyl J., Prozesky L., Stoltsz W.H., Lawrence J.A., Penzhorn B.L., Jongejan F. (2005). Molecular Characterization of *Theileria* Species Associated with Mortality in Four Species of African Antelopes. J. Clin. Microbiol..

[10] Kier A.B., Wightman S.R., Wagner J.E. (1982). Interspecies Transmission of *Cytauxzoon felis*. Am. J. Vet. Res..

[11] Schreeg M.E., Marr H.S., Tarigo J.L., Cohn L.A., Bird D.M., Scholl E.H., Levy M.G., Wiegmann B.M., Birkenheuer A.J. (2016). Mitochondrial Genome Sequences and Structures Aid in the Resolution of Piroplasmida Phylogeny. PLoS ONE.

[12] O’Donoghue P. (2017). Haemoprotozoa: Making Biological Sense of Molecular Phylogenies. Int. J. Parasitol. Parasites Wildl..

[13] Ascencio M., Florin-Christensen M., Mamoun C., Weir W., Shiels B., Schnittger L. (2018). Cysteine Proteinase C1A Paralog Profiles Correspond with Phylogenetic Lineages of Pathogenic Piroplasmids. Vet. Sci..

[14] Wilson R.J., Williamson D.H. (1997). Extrachromosomal DNA in the Apicomplexa. Microbiol. Mol. Biol. Rev..

[15] Tran J.Q., de Leon J.C., Li C., Huynh M.-H., Beatty W., Morrissette N.S. (2010). RNG1 Is a Late Marker of the Apical Polar Ring in *Toxoplasma gondii*. Cytoskeleton.

[16] Sibley L.D. (2010). How Apicomplexan Parasites Move in and out of Cells. Curr. Opin. Biotechnol..

[17] Alexeyev M., Shokolenko I., Wilson G., LeDoux S. (2013). The Maintenance of Mitochondrial DNA Integrity—Critical Analysis and Update. Cold Spring Harb. Perspect. Biol..

[18] Mather M., Henry K., Vaidya A. (2006). Mitochondrial Drug Targets in Apicomplexan Parasites. Curr. Drug Targets.

[19] Schreeg M.E., Marr H.S., Griffith E.H., Tarigo J.L., Bird D.M., Reichard M.V., Cohn L.A., Levy M.G., Birkenheuer A.J. (2016). PCR Amplification of a Multi-Copy Mitochondrial Gene (Cox3) Improves Detection of *Cytauxzoon felis* Infection as Compared to a Ribosomal Gene (18S). Vet. Parasitol..

[20] Kocan A.A., Kocan K.M., Blouin E.F., Mukolwe S.W. (1992). A Redescription of Schizogony of Cytauxzoon Felis in the Domestic Cat. Ann. N. Y. Acad. Sci..

[21] Snider T.A., Confer A.W., Payton M.E. (2010). Pulmonary Histopathology of *Cytauxzoon felis* Infections in the Cat. Vet. Pathol..

[22] Lehane M.J. (1997). Peritrophic Matrix Structure and Function. Annu. Rev. Entomol..

[23] Hegedus D., Erlandson M., Gillott C., Toprak U. (2009). New Insights into Peritrophic Matrix Synthesis, Architecture, and Function. Annu. Rev. Entomol..

[24] Bolognesi R., Terra W.R., Ferreira C. (2008). Peritrophic Membrane Role in Enhancing Digestive Efficiency. Theoretical and Experimental Models. J. Insect Physiol..

[25] Reichard M.V., Edwards A.C., Meinkoth J.H., Snider T.A., Meinkoth K.R., Heinz R.E., Little S.E. (2010). Confirmation of *Amblyomma americanum* (Acari: Ixodidae) as a Vector for Cytauxzoon felis (Piroplasmorida: Theileriidae) to Domestic Cats. J. Med. Entomol..

[26] Thomas J.E., Ohmes C.M., Payton M.E., Hostetler J.A., Reichard M.V. (2018). Minimum Transmission Time of *Cytauxzoon felis* by *Amblyomma americanum* to Domestic Cats in Relation to Duration of Infestation, and Investigation of Ingestion of Infected Ticks as a Potential Route of Transmission. J. Feline Med. Surg..

[27] Allen K.E., Thomas J.E., Wohltjen M.L., Reichard M.V. (2019). Transmission of *Cytauxzoon felis* to Domestic Cats by *Amblyomma americanum* Nymphs. Parasites Vectors.

[28] Nagamori Y., Slovak J.E., Reichard M.V. (2016). Prevalence of *Cytauxzoon felis* Infection in Healthy Free-Roaming Cats in North-Central Oklahoma and Central Iowa. J. Feline Med. Surg. Open Rep..

[29] Holderman C.J., Kaufman P.E. Common Name: Lone Star Tick Scientific Name: Amblyomma americanum (Linnaeus) (Acari: Ixodidae). http://entnemdept.ufl.edu/creatures/urban/medical/lone_star_tick.htm.

[30] Adams D., Anderson B., Ammirati C., Helm K. (2002). Identification And Diseases Of Common U.S. Ticks. Internet J. Dermatol..

[31] Nuttall P.A. (2019). Tick Saliva and Its Role in Pathogen Transmission. Wien. Klin. Wochenschr. Cental Eur. J. Med..

[32] Chmelar J., Kotál J., Kovaríková A., Kotsyfakis M. (2019). The Use of Tick Salivary Proteins as Novel Therapeutics. Front. Physiol..

[33] Chan W.-H., Kaufman P.E. Common Name: American Dog Tick Scientific Name: Dermacentor variabilis (Say) (Arachnida: Ixodida: Ixodidae). http://entnemdept.ufl.edu/creatures/urban/medical/american_dog_tick.htm.

[34] Monzón J.D., Atkinson E.G., Henn B.M., Benach J.L. (2016). Population and Evolutionary Genomics of *Amblyomma americanum*, an Expanding Arthropod Disease Vector. Genome Biol. Evol..

[35] Saleh M.N., Sundstrom K.D., Duncan K.T., Ientile M.M., Jordy J., Ghosh P., Little S.E. (2019). Show Us Your Ticks: A Survey of Ticks Infesting Dogs and Cats across the USA. Parasites Vectors.

[36] Little S.E., Barrett A.W., Nagamori Y., Herrin B.H., Normile D., Heaney K., Armstrong R. (2018). Ticks from Cats in the United States: Patterns of Infestation and Infection with Pathogens. Vet. Parasitol..

[37] Kaufman E.L., Stone N.E., Scoles G.A., Hepp C.M., Busch J.D., Wagner D.M. (2018). Range-Wide Genetic Analysis of *Dermacentor variabilis* and Its *Francisella*-like Endosymbionts Demonstrates Phylogeographic Concordance between Both Taxa. Parasites Vectors.

[38] Minigan J.N., Hager H.A., Peregrine A.S., Newman J.A. (2018). Current and Potential Future Distribution of the American Dog Tick (*Dermacentor variabilis*, Say) in North America. Ticks Tick. Borne. Dis..

[39] Blouin E.F., Kocan A.A., Glenn B.L., Kocan K.M., Hair J.A. (1984). Transmission of *Cytauxzoon felis* Kier, 1979 from Bobcats, *Felis rufus* (Schreber), to Domestic Cats by *Dermacentor veriabilis* (Say). J. Wildl. Dis..

[40] Reichard M.V., Meinkoth J.H., Edwards A.C., Snider T.A., Kocan K.M., Blouin E.F., Little S.E. (2009). Transmission of *Cytauxzoon felis* to a Domestic Cat by *Amblyomma americanum*. Vet. Parasitol..

[41] Glenn B.L., Kocan A.A., Blouin E.F. (1983). Cytauxzoonosis in Bobcats. J. Am. Vet. Med. Assoc..

[42] Blouin E.F., Kocan A.A., Kocan K.M., Hair J. (1987). Evidence of a Limited Schizogonous Cycle for *Cytauxzoon felis* in Bobcats Following Exposure to Infected Ticks. J. Wildl. Dis..

[43] Birkenheuer A.J., Marr H.S., Warren C., Acton A.E., Mucker E.M., Humphreys J.G., Tucker M.D. (2008). *Cytauxzoon felis* Infections Are Present in Bobcats (*Lynx rufus*) in a Region Where Cytauxzoonosis Is Not Recognized in Domestic Cats. Vet. Parasitol..

[44] Zieman E.A., Nielsen C.K., Jiménez F.A. (2018). Chronic *Cytauxzoon felis* Infections in Wild-Caught Bobcats (*Lynx rufus*). Vet. Parasitol..

[45] Shock B.C., Murphy S.M., Patton L.L., Shock P.M., Olfenbuttel C., Beringer J., Prange S., Grove D.M., Peek M., Butfiloski J.W. (2011). Distribution and Prevalence of *Cytauxzoon felis* in Bobcats (*Lynx rufus*), the Natural Reservoir, and Other Wild Felids in Thirteen States. Vet. Parasitol..

[46] Brown H.M., Lockhart J.M., Latimer K.S., Peterson D.S. (2010). Identification and Genetic Characterization of *Cytauxzoon felis* in Asymptomatic Domestic Cats and Bobcats. Vet. Parasitol..

[47] Lewis K.M., Cohn L.A., Marr H.S., Birkenheuer A.J. (2012). Diminazene Diaceturate for Treatment of Chronic *Cytauxzoon felis* Parasitemia in Naturally Infected Cats. J. Vet. Intern. Med..

[48] Butt M.T., Bowman D., Barr M.C., Roelke M.E. (1991). Iatrogenic Transmistion of *Cytauxzoon felis* from a Florida Panther (*Felix concolor coryi*) to a Domestic Cat. J. Wildl. Dis..

[49] Rotstein D.S., Taylor S.K., Harvey J.W., Bean J. (1999). Hematologic Effects of Cytauxzoonosis in Florida Panthers and Texas Cougars in Florida. J. Wildl. Dis..

[50] Harvey J.W., Dunbar M.R., Norton T.M., Yabsley M.J. (2007). Laboratory Findings in Acute *Cytauxzoon felis* Infection in Cougars (*Puma concolor couguar*) in Florida. J. Zoo Wildl. Med..

[51] Lewis K.M., Cohn L.A., Downey M.E., Whitney M.S., Birkenheuer A.J. (2012). Evaluation of *Cytauxzoon felis* Infection Status in Captive-Born Wild Felids Housed in an Area Endemic for the Pathogen. J. Am. Vet. Med. Assoc..

[52] Cerreta A.J., Yang T.S., Ramsay D.C., Birkenheuer A.J., Rahoi D., Qurollo B., Wilsn J., Cushing A.C. (2022). Detection of Vector-borne Infections in Lions and Tigers at Two Zoos in Tennessee and Oklahoma, USA. J. Zoo Wildl. Med..

[53] Rizzi T.E., Reichard M.V., Cohn L.A., Birkenheuer A.J., Taylor J.D., Meinkoth J.H. (2015). Prevalence of *Cytauxzoon felis* Infection in Healthy Cats from Enzootic Areas in Arkansas, Missouri, and Oklahoma. Parasites Vectors.

[54] Nietfeld J.C., Pollock C. (2002). Fatal Cytauxzoonosis in a Free-Ranging Bobcat (*Lynx rufus*). J. Wildl. Dis..

[55] Hoover J.P., Walker D.B., Hedges J.D. (1994). Cytauxzoonosis in Cats: Eight Cases (1985–1992). J. Am. Vet. Med. Assoc..

[56] Meinkoth J.H., Kocan A.A. (2005). Feline Cytauxzoonosis. Vet. Clin. N. Am. Small Anim. Pract..

[57] Meinkoth J., Kocan A.A., Whitworth L., Murphy G., Fox J.C., Woods J.P. (2000). Cats Surviving Natural Infection with *Cytauxzoon felis*: 18 Cases (1997-1998). J. Vet. Intern. Med..

[58] Jackson C.B., Fisher T. (2006). Fatal Cytauxzoonosis in a Kentucky Cat (Felis Domesticus). Vet. Parasitol..

[59] Haber M.D., Tucker M.D., Marr H.S., Levy J.K., Burgess J., Lappin M.R., Birkenheuer A.J. (2007). The Detection of *Cytauxzoon felis* in Apparently Healthy Free-Roaming Cats in the USA. Vet. Parasitol..

[60] Brown H.M., Latimer K.S., Erikson L.E., Cashwell M.E., Britt J.O., Peterson D.S. (2008). Detection of Persistent *Cytauxzoon felis* Infection by Polymerase Chain Reaction in Three Asymptomatic Domestic Cats. J. Vet. Diagn. Investig..

[61] MacNeill A.L., Barger A.M., Skowronski M.C., Lanka S., Maddox C.W. (2015). Identification of *Cytauxzoon felis* Infection in Domestic Cats from Southern Illinois. J. Feline Med. Surg..

[62] Reichard M.V., Baum K.A., Cadenhead S.C., Snider T.A. (2008). Temporal Occurrence and Environmental Risk Factors Associated with Cytauxzoonosis in Domestic Cats. Vet. Parasitol..

[63] Birkenheuer A.J., Le J.A., Valenzisi A.M., Tucker M.D., Levy M.G., Breitschwerdt E.B. (2006). *Cytauxzoon felis* Infection in Cats in the Mid-Atlantic States: 34 Cases (1998–2004). J. Am. Vet. Med. Assoc..

[64] Kao Y., Peake B., Madden R., Cowan S.R., Scimeca R.C., Thomas J.E., Reichard M.V., Ramachandran A., Miller C.A. (2021). A probe-based droplet digital polymerase chain reaction assay for early detection of feline acute cytauxzoonosis. J. Vet. Parasit..

[65] Frontera-Acevedo K. (2013). Feline Immune Response To Infection With *Cytauxzoon felis* and the Role of CD18 in the Pathogenesis of Cytauxzoonosis. Ph.D. Thesis.

[66] Frontera-Acevedo K., Sakamoto K. (2015). Local Pulmonary Immune Responses in Domestic Cats Naturally Infected with *Cytauxzoon felis*. Vet. Immunol. Immunopathol..

[67] Ridgway M.D. Feline Cytauxzoonosis. https://vetmed.illinois.edu/wp-content/uploads/2015/08/22.-Feline-Cytauxzoonosis.pdf.

[68] Conner B.J., Hanel R.M., Brooks M.B., Cohn L.A., Birkenheuer A.J. (2015). Coagulation Abnormalities in 5 Cats with Naturally Occurring Cytauxzoonosis. J. Vet. Emerg. Crit. Care.

[69] Clarke L.L., Rissi D.R. (2015). Neuropathology of Natural *Cytauxzoon felis* Infection in Domestic Cats. Vet. Pathol..

[70] Clarke L.L., Krimer P.M., Rissi D.R. (2017). Glial Changes and Evidence for Apoptosis in the Brain of Cats Infected by *Cytauxzoon felis*. J. Comp. Pathol..

[71] Cohn L.A., Shaw D., Shoemake C., Birkenheuer A.J. (2020). Second Illness Due to Subsequent *Cytauxzoon felis* Infection in a Domestic Cat. J. Feline Med. Surg. Open Rep..

[72] Lewis K. (2011). *Cytauxzoon felis*: An Emerging Feline Pathogen and Potential Therapy.

[73] Sleznikow C.R., Granick J.L., Cohn L.A., Nafe L.A., Rendahl A., Burton E.N. (2021). Evaluation of various sample sources for the cytologic diagnosis of *Cytauxzoon felis*. J. Vet. Int. Med..

[74] Sykes J.E. (2010). Feline Hemotropic Mycoplasmas. Vet. Clin. N. Am. Small Anim. Pract..

[75] Penzhorn B.L., Oosthuizen M.C. (2020). *Babesia* Species of Domestic Cats: Molecular Characterization Has Opened Pandora’s Box. Front. Vet. Sci..

[76] Motzel S.L., Wagner J.E. (1990). Treatment of Experimentally Induced Cytauxzoonosis in Cats with Parvaquone and Buparvaquone. Vet. Parasitol..

[77] Brown H.M., Modaresi S.M., Cook J.L., Latimer K.S., Peterson D.S. (2009). Genetic Variability of Archived *Cytauxzoon felis* Histologic Specimens from Domestic Cats in Georgia, 1995-2007. J. Vet. Diagn. Investig..

[78] Lewis K.M., Cohn L.A., Marr H.S., Birkenheuer A.J. (2014). Failure of Efficacy and Adverse Events Associated with Dose-Intense Diminazene Diaceturate Treatment of Chronic *Cytauxzoon felis* Infection in Five Cats. J. Feline Med. Surg..

[79] Cohn L.A., Birkenheuer A.J., Brunker J.D., Ratcliff E.R., Craig A.W. (2011). Efficacy of Atovaquone and Azithromycin or Imidocarb Dipropionate in Cats with Acute Cytauxzoonosis. J. Vet. Intern. Med..

[80] Vaidya A.B., Mather M.W. (2009). Mitochondrial Evolution and Functions in Malaria Parasites. Annu. Rev. Microbiol..

[81] Schreeg M.E., Marr H.S., Tarigo J., Cohn L.A., Levy M.G., Birkenheuer A.J. (2013). Pharmacogenomics of *Cytauxzoon felis* Cytochrome b: Implications for Atovaquone and Azithromycin Therapy in Domestic Cats with Cytauxzoonosis. J. Clin. Microbiol..

[82] Schreeg M.E., Marr H.S., Tarigo J.L., Cohn L.A., Levy M.G., Birkenheuer A.J. (2015). Rapid High-Resolution Melt Analysis of *Cytauxzoon felis* Cytochrome b to Aid in the Prognosis of Cytauxzoonosis. J. Clin. Microbiol..

[83] Srivastava I.K., Rottenberg H., Vaidya A.B. (1997). Atovaquone, a Broad Spectrum Antiparasitic Drug, Collapses Mitochondrial Membrane Potential in a Malarial Parasite. J. Biol. Chem..

[84] Hikosaka K., Komatsuya K., Suzuki S., Kira K. (2015). Mitochondria of Malaria Parasites as a Drug Target. An Overview of Tropical Diseases.

[85] Tarigo J. (2013). The *Cytauxzoon felis* Genome: A Guide to Vaccine Candidate Antigen Discovery for Cytauxzoonosis. Ph.D. Thesis.

[86] Pollard D.A., Reichard M.V., Cohn L.A., James A.M., Holman P.J. (2017). Genetic Variability of Cloned *Cytauxzoon felis* Ribosomal RNA ITS1 and ITS2 Genomic Regions from Domestic Cats with Varied Clinical Outcomes from Five States. Vet. Parasitol..

[87] Tarigo J.L., Scholl E.H., Bird D.M.K., Brown C.C., Cohn L.A., Dean G.A., Levy M.G., Doolan D.L., Trieu A., Nordone S.K. (2013). A Novel Candidate Vaccine for Cytauxzoonosis Inferred from Comparative Apicomplexan Genomics. PLoS ONE.

[88] Tarigo J.L., Kelly L.S., Brown H.M., Peterson D.S. (2019). Limited Genetic Variability of *Cytauxzoon felis* Apical Membrane Antigen-1 (Ama1) from Domestic Cats and Bobcats. Parasites Vectors.

[89] Reichard M.V., Thomas J.E., Arther R.G., Hostetler J.A., Raetzel K.L., Meinkoth J.H., Little S.E. (2013). Efficacy of an Imidacloprid 10%/Flumethrin 4.5% Collar (Seresto^®^, Bayer) for Preventing the Transmission of *Cytauxzoon felis* to Domestic Cats by *Amblyomma americanum*. Parasitol. Res..

[90] Reichard M.V., Rugg J.J., Thomas J.E., Allen K.E., Barrett A.W., Murray J.K., Herrin B.H., Beam R.A., King V.L., Vatta A.F. (2019). Efficacy of a Topical Formulation of Selamectin plus Sarolaner against Induced Infestations of *Amblyomma americanum* on Cats and Prevention of *Cytauxzoon felis* Transmission. Vet. Parasitol..

